# Atrial Fibrillation-Linked Germline *GJA5*/Connexin40 Mutants Showed an Increased Hemichannel Function

**DOI:** 10.1371/journal.pone.0095125

**Published:** 2014-04-14

**Authors:** Yiguo Sun, Matthew D. Hills, Willy G. Ye, Xiaoling Tong, Donglin Bai

**Affiliations:** Department of Physiology and Pharmacology, The University of Western Ontario, London, Ontario, Canada; Gent University, Belgium

## Abstract

Mutations in *GJA5* encoding the gap junction protein connexin40 (Cx40) have been linked to lone atrial fibrillation. Some of these mutants result in impaired gap junction function due to either abnormal connexin localization or impaired gap junction channels, which may play a role in promoting atrial fibrillation. However, the effects of the atrial fibrillation-linked Cx40 mutants on hemichannel function have not been studied. Here we investigated two atrial fibrillation-linked germline Cx40 mutants, V85I and L221I. These two mutants formed putative gap junction plaques at cell-cell interfaces, with similar gap junction coupling conductance as that of wild-type Cx40. Connexin deficient HeLa cells expressing either one of these two mutants displayed prominent propidium iodide-uptake distinct from cells expressing wild-type Cx40 or other atrial fibrillation-linked Cx40 mutants, I75F, L229M, and Q49X. Propidium iodide-uptake was sensitive to [Ca^2+^]_o_ and the hemichannel blockers, carbenoxolone, flufenamic acid and mefloquine, but was not affected by the pannexin 1 channel blocking agent, probenecid, indicating that uptake is most likely mediated via connexin hemichannels. A gain-of-hemichannel function in these two atrial fibrillation-linked Cx40 mutants may provide a novel mechanism underlying the etiology of atrial fibrillation.

## Introduction

Gap junctions are intercellular channels formed by dodecamers of integral membrane protein subunits known as connexins (Cxs). Gap junctions allow direct exchange of ions and small molecules between apposing cells [1]. The Cx family of proteins all share a common structural topology, which consists of an intracellular amino-terminus, four transmembrane domains, two extracellular loops, a cytoplasmic loop and an intracellular carboxyl-terminus [2]. The oligomerization of six Cxs forms a hemichannel (also known as connexon) and two hemichannels on the plasma membrane of neighbouring cells can dock end-to-end to form a gap junction channel.

In addition to forming gap junction channels, Cxs are able to form undocked hemichannels on the plasma membrane. These hemichannels can provide a direct passage between the intracellular environment and the extracellular space, which allows for the release of small intracellular molecules such as ATP [3], glutamate [4], NAD^+^ [5] and prostaglandin E2 [6]. These signaling molecules can then act on their respective receptors located on the same cell (autocrine) or its neighbouring cells (paracrine). A common feature of all hemichannels is that under physiological conditions they have a low open probability, but can be opened by a number of different stimuli including reduced concentrations of extracellular divalent cations, such as Ca^2+^ and Mg^2+^, large and prolonged membrane depolarization, mechanical membrane stress and/or metabolic inhibition [7], [8].

In the heart, gap junctions mediate direct electrical coupling between cardiomyocytes, allowing for rapid propagation of action potentials in the atria and ventricles, which is essential for synchronous contractions [9]. The human heart expresses three main Cx isoforms: Cx40, Cx43 and Cx45. Both Cx40 and Cx43 are expressed in the atria and Cx43 is the major connexin in the ventricles. In contrast, Cx45 is mainly found in the sinoatrial and atrioventricular nodes [10]. In addition to its extensive expression in the atria, Cx40 is also found in parts of the ventricular conduction system, such as the His-bundle, the upper and lower bundle branches and the Purkinje fibres. Several recent studies indicate somatic and germline mutations in the Cx40 gene (*GJA5*) are associated with lone atrial fibrillation (AF) [11], [12], [13], [14], [15]. Studies by us and others on these AF-linked Cx40 mutants revealed various changes in cellular distribution and gap junction function [11], [12], [15], [16]. However, it is not known if there are any changes in the hemichannel function for any of the AF-linked Cx40 mutants. Here we investigated two novel germline Cx40 mutations, V85I and L221I, identified by Yang et al. (2010b) from two Chinese families with inherited lone AF. These mutations were autosomal dominantly inherited and mutant carriers in the family showed early onset of AF. These mutations were not found in other members in the family or in 200 unrelated, healthy, ethnic- and age-matched control subjects [13]. We observed little change in the gap junction distribution and function of these two Cx40 mutants in HeLa and N2A cells. However, these two AF-linked Cx40 mutants showed an increase in propidium iodide (PI) uptake under conditions favoring hemichannel opening. Interestingly, we did not observe any PI-uptake in wild-type Cx40 expressing cells, indicating that the mutants showed a gain-of-hemichannel function, which may play a role in the pathogenesis of AF.

## Methods

### Plasmid Construction

The human Cx40-YFP, Cx40-IRES-GFP, Cx43-IRES-GFP and Cx26-GFP constructs were created as previously described [12], [17]. The C-terminal fusion YFP-tagged (V85I-YFP and L221I-YFP) and the non-fusion GFP-tagged (V85I-IRES-GFP and L221I-IRES-GFP) constructs were generated by the Quick-Change site directed mutagenesis kit (Stratagene, La Jolla, CA) on the respective template with the following primers: the forward 5′-CAGATCATCTTCATCTCCACGCCCT-3′ and the reverse 5′-AGGGCGTGGAGATGAAGATGATCTG-3′ for V85I and the forward 5′-CTGTCCCTCCTCATTAGCCTGGCTG-3′ and the reverse 5′-CAGCCAGGCTAATGAGGAGGGACAG-3′ for L221I. All connexin clones were sequenced to confirm the accuracy of the nucleotide sequence and no additional variations were introduced.

### Cell Culture and Transfection

HeLa (human cervical carcinoma, American Type Culture Collection, Manassas, VA) cells were grown in Dulbecco’s modified Eagle’s medium (DMEM, Invitrogen, Burlington, ON) containing 4.5 g/L D-glucose, 584 mg/L L-glutamine, 110 mg/L sodium pyruvate, 10% fetal bovine serum and 1% penicillin and streptomycin, in an incubator with 5% CO_2_ at 37°C. HeLa cells were plated at 60–80% confluence on 35 mm Petri dishes 12–24 hours before transfection. For each transfection, HeLa cells were incubated with 1.5 µg of a cDNA construct and 3 µl of X-tremeGENE HP DNA transfection reagent (Roche, Mississauga, ON) in Opti-MEM I+GlutaMAX-I medium supplemented with HEPES and 2.4 g/L sodium bicarbonate (Invitrogen) for 4 hours. Medium was then changed back to the modified DMEM and cells were used for either localization studies or dye uptake assays approximately 18–24 hours after transfection.

### Localization Study

To observe the localization of Cx40-YFP, V85I-YFP and L221I-YFP, HeLa cells were cultured on glass bottom dishes and were transfected individually with the respective cDNA constructs. After culturing for 24 hours, the cells were fixed with a solution of 80% methanol and 20% acetone for 20 minutes at −20°C. Wild-type Cx40-YFP and YFP-tagged mutants were imaged using a Zeiss LSM 510-META confocal microscope as described earlier [12]. To quantify the percentage of gap junction plaque-like structures at the cell-to-cell interfaces of successfully transfected cells, approximately 20–30 cells were counted for each transfection.

To observe the localization of untagged Cx40 and mutants, HeLa and N2A cells (American Type Culture Collection) were transfected with Cx40-IRES-GFP, V85I-IRES-GFP or L221I-IRES-GFP. After culturing for 24 h, cells were rinsed with PBS and fixed for 10 minutes in a 1∶1 solution of acetone and methanol at −20°C. Cells were then blocked for 1 hour with 5% BSA in PBS. Anti-Cx40 antibody (Millipore, Billerica, MA) was incubated for 1 h at room temperature. The cells were washed and subsequently stained for 30 min with the secondary Alexa 594–conjugated antibody (Invitrogen) prior to confocal microscopy.

### Dye Uptake Assay

Propidium iodide (PI)-uptake assay was used to assess the hemichannel function of YFP-tagged Cx40 and mutants. HeLa cells were plated at a low density to allow for isolated, single cells to be transiently transfected as described above. The cells were washed with regular extracellular solution (ECS) (also known as divalent cation-containing ECS, DCC-ECS) containing 142 mM NaCl, 5.4 mM KCl, 1.4 mM MgCl_2_, 2 mM CaCl_2_, 10 mM HEPES and 25 mM D-Glucose. The pH of ECS was adjusted to 7.35 and the osmolarity was adjusted to 298 mOsm. The cells were then washed with divalent cation free-ECS (DCF-ECS), which contains no Ca^2+^ or Mg^2+^ and 2 mM EGTA to chelate the remaining ambient divalent cations. The cells were incubated in DCF-ECS-containing PI (150 µM) at 37°C for 15 minutes to assess PI-uptake. After incubation, the cells were washed three times with regular ECS and the percentage of transfected cells (green with either GFP or YFP) showing PI-uptake was measured under a fluorescent microscope (DMIRE2, Leica). Cells in pairs and clusters were excluded from measurement to avoid errors produced by gap junctions. Negative controls (untransfected cells and YFP-transfected cells) and positive control (Cx26-GFP transfected cells) and the various incubation conditions (e.g. with hemichannel blockers carbenoxolone, flufenamic acid, mefluquine or different [Ca^2+^]_o_) were indicated in each experiment. For each experiment approximately 30–50 cells were counted to obtain a percentage of PI-uptake. The bar graphs were generated with 5–15 transfections. Similar PI-uptake experiments were performed on HeLa cells transfected with the mutant-IRES-GFP constructs with a slightly longer PI-incubation time (20 minutes).

For the experiment with continuous measurement of PI-uptake, HeLa cells were cultured in glass bottom dishes. Fluorescence measurements of PI were performed with a confocal microscope (LSM 510 Meta, Zeiss, Germany). Baseline fluorescence (F0) in regular ECS and the increase of PI-uptake during the incubation of DCF-ECS (F) were collected at 1 minute intervals for 20 minutes. The obtained images were quantitatively analyzed using ZEN software for changes in fluorescence intensities within regions of interest (ROIs) of isolated GFP-positive cells, which expressed mutant-IRES-GFP. The intracellular fluorescence changes during PI incubation are expressed as the ratio of current fluorescence intensity over that of the baseline (F/F_0_).

### Electrophysiological Studies

Electrophysiological recordings for measuring gap junction coupling were carried out in connexin-deficient neuroblastoma (N2A) cells. N2A cells were grown at 37°C in 35-mm culture dishes to 70% confluence in Dulbecco’s modified Eagle’s medium containing 10% FBS. Cells were transiently transfected with mutant or wild-type connexin DNA by X-tremeGENE HP reagent. The dual whole-cell patch clamp technique was performed at room temperature to assess the gap junctional conductance (G_j_) between cell pairs 24 hours after transfection [12], [18]. For co-transfection experiments, cell pairs showing successful co-expression with V85I-IRES-GFP (or L221I-GFP) and Cx43mRFP were selected for recording as described earlier [12]. The junctional current (I_j_) was amplified via a MultiClamp 700A amplifier (Molecular Devices, Sunnyvale, CA) and was digitized at a sampling rate of 10 kHz with a Digidata 1322A (Molecular Devices, Sunnyvale, CA). Data were analyzed with pClamp9 software. Each cell of a pair was initially held at a common holding potential of 0 mV. To evaluate junctional coupling, 20 mV pulses for 7 seconds were applied to one cell to establish a transjunctional voltage (V_j_), while the junctional currents (I_j_) were measured in the other cell. Macroscopic junctional conductance (G_j_) was calculated as follows: G_j_ = I_j_/V_j_. In all cases, cells were studied after multiple independent transfections and only cell pairs with fluorescent protein signals were selected for double patch clamp recording.

Hemichannel currents were studied in the mutant and control connexins (all with untagged GFP in pIRES2-GFP vector) transfected HeLa cells using a voltage ramp protocol (from −110 to +110 mV in 6 seconds, 36.7 mV/s) similar to that previously reported [19]. DCF-ECS was used to facilitate the hemichannel opening. Pipette solution and extracellular saline were the same as those described earlier [12]. Carbenoxolone (CBX 100 µM) was used to block the hemichannel current.

### Statistical Analysis

One-way ANOVA followed by Newman-Keuls test was used to compare the multiple groups of data on G_j_ and PI-uptake percentage. Statistical significance is denoted with asterisks (*, *P*<0.05 or ***, *P*<0.001) on the graphs. The data presented on the graphs are expressed as mean ± standard error of the mean (SEM). Unless specified, all experiments were performed at least three times.

## Results

### Localization of YFP-tagged Cx40 Mutants

**Figure 1:**
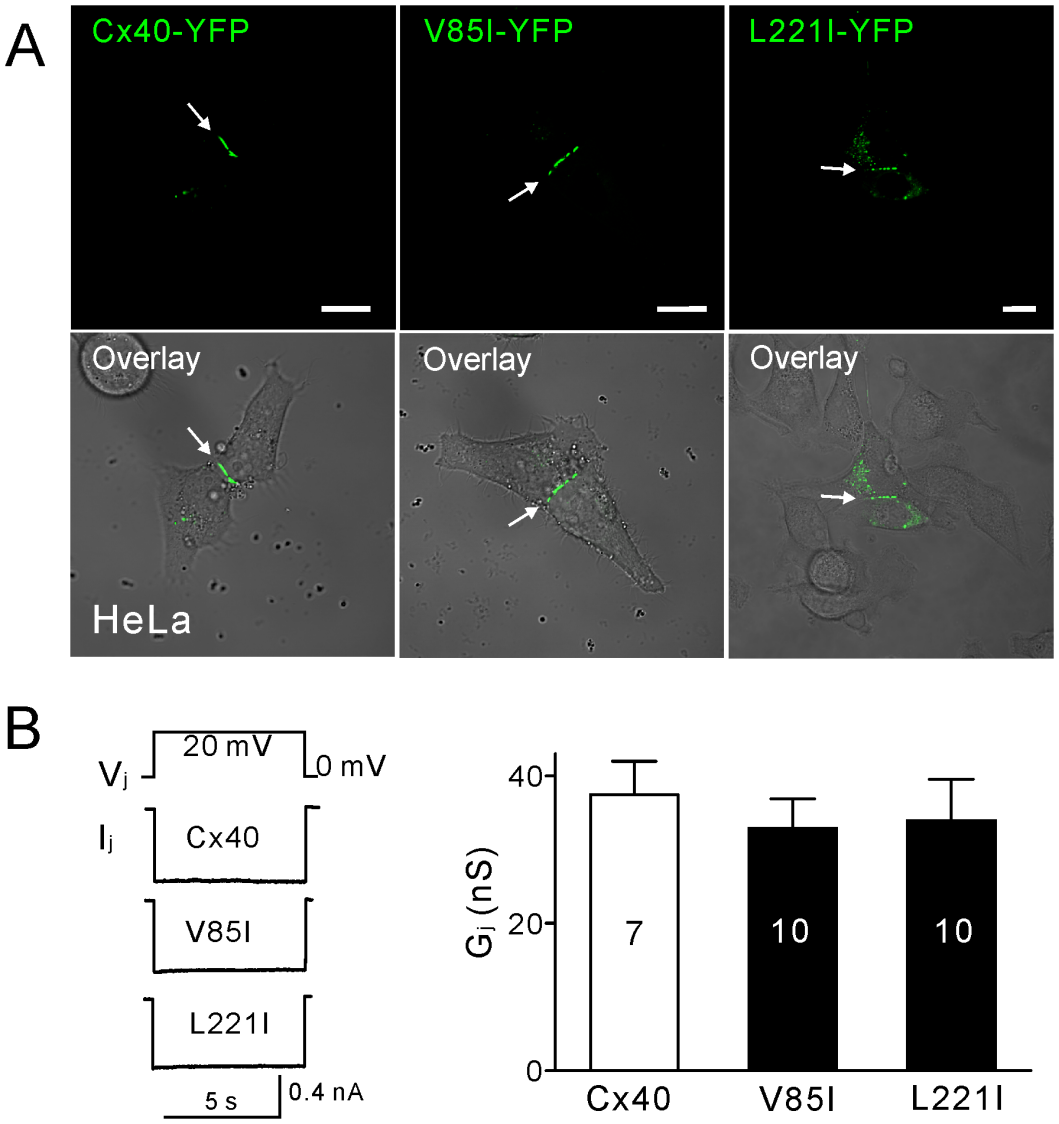
The localization and macroscopic dual whole-cell patch clamp recordings of YFP-tagged homotypic Cx40 and Cx40-mutant gap junctions. (**A**) Representative fluorescent confocal images of Cx40-YFP, V85I-YFP and L221I-YFP (top panels). The overlaid fluorescent images on top of phase contrast images are also shown (bottom panels). All three constructs were able to form gap junction plaque-like structures at the cell-cell junction. Scale bar = 10 µm. (**B**) Voltage steps of 20 mV were applied to one cell of a transfected N2A cell pair and the junctional current (I_j_) was recorded in the second cell. There was no significant difference between the I_j_ in cell pairs expressing Cx40-YFP, V85I-YFP or L221I-YFP. The junctional conductance (G_j_) was calculated and there was no significant difference between the G_j_ of cell pairs expressing Cx40-YFP, V85I-YFP or L221I-YFP.

The localization of wild-type Cx40-YFP and the AF-linked Cx40 mutants, V85I-YFP or L221I-YFP, were examined in connexin-deficient HeLa cells. As shown in Fig. 1A, Cx40-YFP, V85I-YFP and L221I-YFP were all able to traffic to the plasma membrane and form gap junction plaque-like structures at cell-cell interfaces. Free YFP did not form gap junction plaque-like structures at cell-cell interfaces (data not shown). To further quantify the probability of gap junction plaque formation, we calculated the percentage of the cell pairs/clusters displaying putative gap junction plaques at cell-cell interfaces. The percentage of successful formation of gap junction plaques of V85I-YFP- and L221I-YFP-expressing cells were 65±2% (n = 7) and 46±2% (n = 7), respectively and were found to be statistically lower than that of the cells expressing Cx40-YFP (89±2%, n = 7; *P*<0.001 for both mutants), indicating that these two AF-linked mutants showed a modest but statistically significant decrease in the formation of gap junction plaque-like structures at cell-cell interfaces.

### G_j_s of the V85I- and L221I-expressing Cell Pairs were the Same as that of Cell Pairs Expressing Wild-type Cx40

Dual patch clamp technique was used to measure the coupling conductance (G_j_) of N2A cell pairs expressing Cx40-YFP, V85I-YFP or L221I-YFP. Transjunctional currents (I_j_s) in response to a 20 mV transjunctional voltage pulse (V_j_) are shown in Fig. 1B. Our results indicate that the averaged coupling conductance (G_j_s) for each mutant was not statistically different from the control (Cx40), demonstrating that the gap junction function of these two mutants was not impaired in the N2A cells.

### V85I- and L221I-expressing Cells Showed Increased Propidium Iodide-uptake

**Figure 2:**
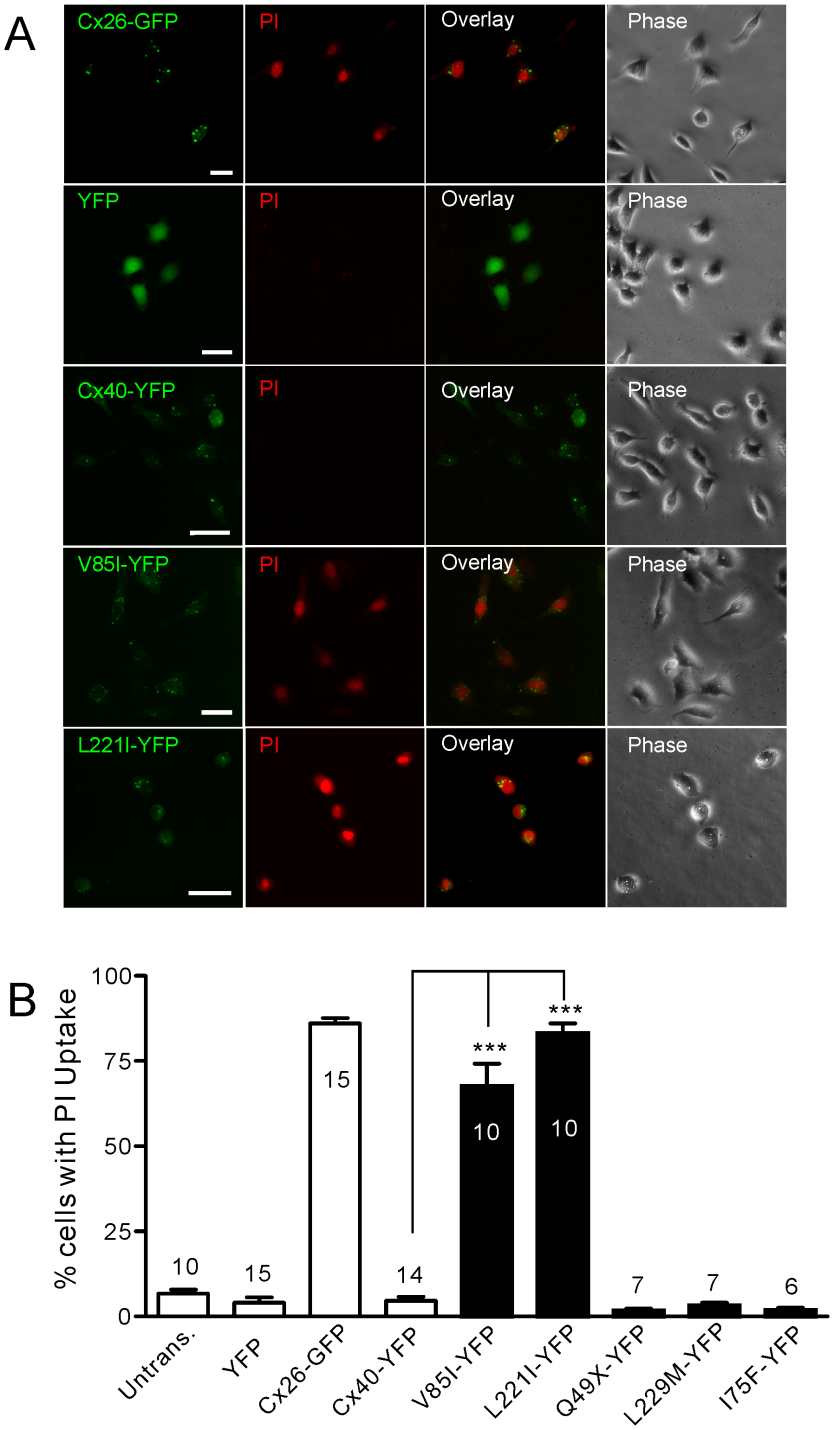
Propidium iodide-uptake under divalent cation-free conditions. (**A**) Representative images of propidium iodide (PI)-uptake under divalent cation-free (DCF) conditions for isolated, individual, transfected HeLa cells. Successful transfection can be identified by their tagged green/yellow fluorescent proteins (green colour in the first column images). PI-uptake (red colour in the second column images) can be seen in cells expressing Cx26-GFP, V85I-YFP and L221I-YFP, but no uptake was seen in cells expressing YFP alone or Cx40-YFP. Scale bar = 20 µm. (**B**) Quantification of PI-uptake under DCF conditions. V85I-YFP (67.6%, n = 10) and L221I-YFP (83.2%, n = 10) showed a significant increase in PI-uptake compared to Cx40-YFP (4.6%, n = 14, ***indicates P<0.001). Other AF-linked Cx40 mutants, Q49X, L229M and I75F, were also studied and did not show any PI-uptake.

Since we observed no apparent gap junction function defects of these two Cx40 mutants in our model cells, this prompted us to look into possible changes in non-gap junction linked functions, including hemichannel function. To facilitate the opening of undocked Cx40 gap junction hemichannels in HeLa cells, we removed both Ca^2+^ and Mg^2+^ and added EGTA (2 mM) to chelate the ambient low level of divalent cations. This solution was defined as divalent cation free-extracellular solution (DCF-ECS or DCF). HeLa cells expressing Cx26-GFP, incubated in DCF-ECS and PI, showed a prominent PI-uptake in 86% of cells (Fig. 2A, B), while the majority of untransfected HeLa cells or YFP-expressing HeLa cells failed to show PI-uptake (Fig. 2A, B), suggesting that undocked Cx26 hemichannels may be responsible for the PI-uptake. Interestingly, positive PI-uptake was identified in the majority of HeLa cells expressing YFP-tagged Cx40 mutants, V85I (67.6±6.6%, n = 10) and L221I (83.2±2.8%, n = 10). In contrast to these findings, Cx40-YFP-expressing cells failed to show a significant PI-uptake under the same conditions (4.6±1.2%, n = 14). This was significantly different from the PI-uptake observed for the two mutants (*P*<0.001), but was similar to that of the negative control, YFP-expressing cells (4.0±1.6%, *P*>0.05).

Our previous studies showed that AF-linked Cx40 mutants, I75F and Q49X, impaired homotypic gap junction function, while L229M did not impair homotypic gap junction function, but specifically impaired the gap junction function when co-expressed with Cx43 [12], [15]. Here we tested PI-uptake of HeLa cells expressing these Cx40 mutants individually. As shown in Fig. 2B, these mutants all failed to show any substantial PI-uptake, indicating that either their undocked hemichannels were unlikely to be in the open state during the incubation with DCF-ECS or in the case of Q49X, it is probably unable to oligomerize to form hemichannels and even if it could form hemichannels, they would be unlikely to reach the plasma membrane.

### The Role of Extracellular Divalent Cations and Carbenoxolone on PI-uptake

**Figure 3:**
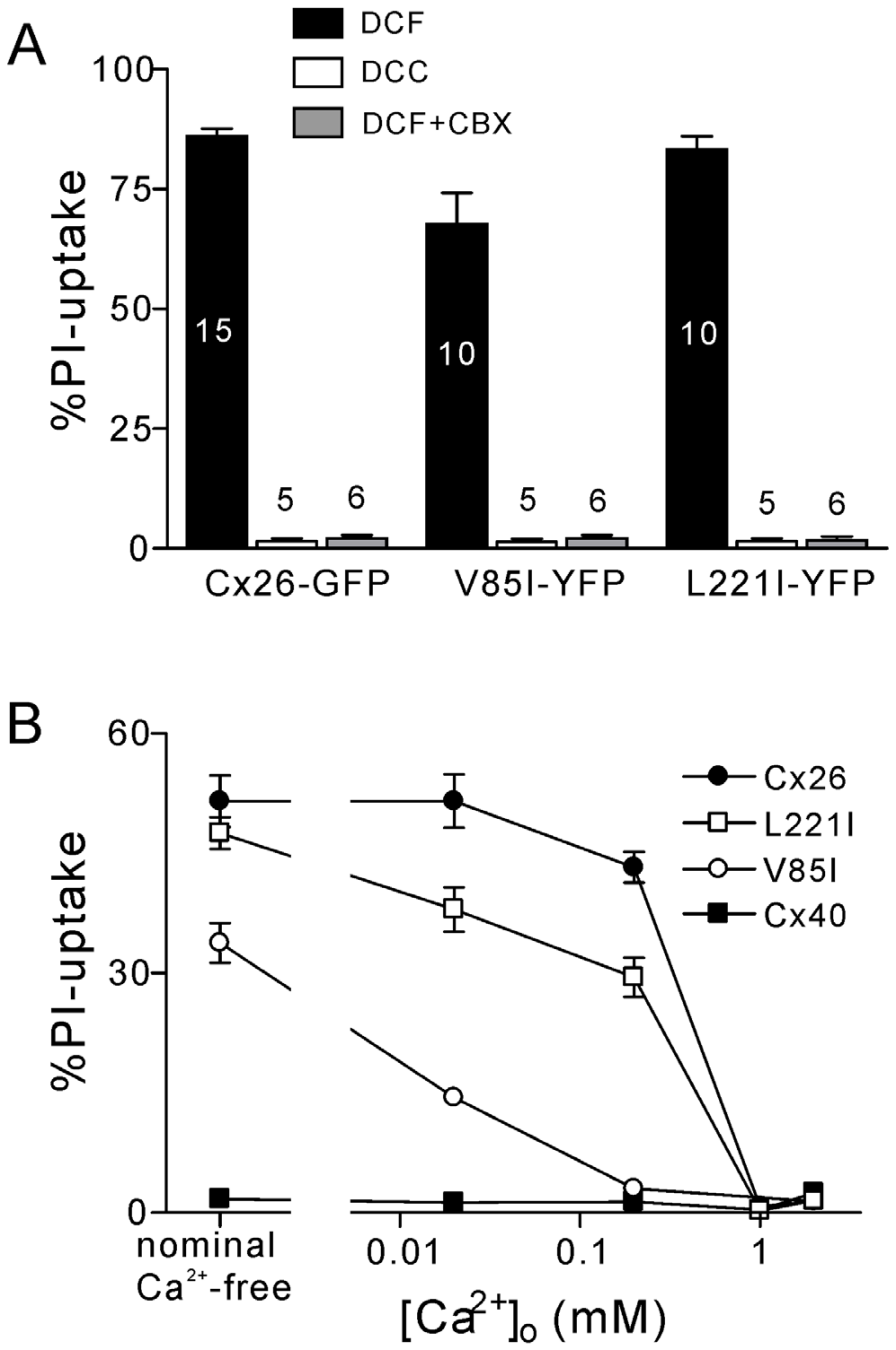
The effect of external Ca^2+^ concentration on PI-uptake. (**A**) Comparison of PI-uptake for divalent cation containing (DCC) and divalent cation-free (DCF) conditions. The PI-uptake for cells expressing Cx26-GFP, V85I-YFP and L221I-YFP was significantly increased under DCF conditions compared to DCC conditions. Also the addition of the hemichannel blocker carbenoxolone (CBX, 100 µM) under DCF conditions significantly decreased PI-uptake. (**B**) [Ca^2+^]_o_ dose-dependent PI-uptake. Cx26-GFP, V85I-YFP and L221I-YFP all showed [Ca^2+^]_o_ dependent PI-uptake. Cx40-YFP did not show PI-uptake for any concentrations tested.

Previous studies indicated that several gap junction hemichannel-mediated dye-uptake could be blocked by the elevation of extracellular calcium concentration or addition of the hemichannel blocker, carbenoxolone (CBX) [8]. We hypothesized that PI-uptake was due to the undocked connexin hemichannels on the plasma membrane. To test this, transfected HeLa cells were incubated with PI in the presence of divalent cation containing solution (DCC-ECS or DCC) or CBX (100 µM). Both DCC-ECS and CBX effectively eliminated PI-uptake in cells expressing Cx26-GFP and the Cx40 mutants, V85I and L221I (Fig. 3A).

To quantitatively assess [Ca^2+^]_o_ dependence of the PI-uptake, several [Ca^2+^]_o_ concentrations from nominal Ca^2+^-free to 2 mM were tested. Our data indicated that cells expressing Cx26 or the Cx40 mutants, V85I and L221I, displayed [Ca^2+^]_o_ concentration-dependent PI-uptake (Fig. 3B). In the range of 0.2 and 0.02 mM [Ca^2+^]_o_, Cx26-expressing cells showed a higher level, L221I-expressing cells showed an intermediate level and V85I-expressing cells showed a lower level of PI-uptake (Fig. 3B), suggesting that these hemichannels may have different sensitivities to [Ca^2+^]_o_. For Cx40-expressing cells, no PI-uptake was observed for any of the calcium concentrations tested (Fig. 3B).

### Hemichannel Characterizations Using Untagged Cx40 Mutants

**Figure 4:**
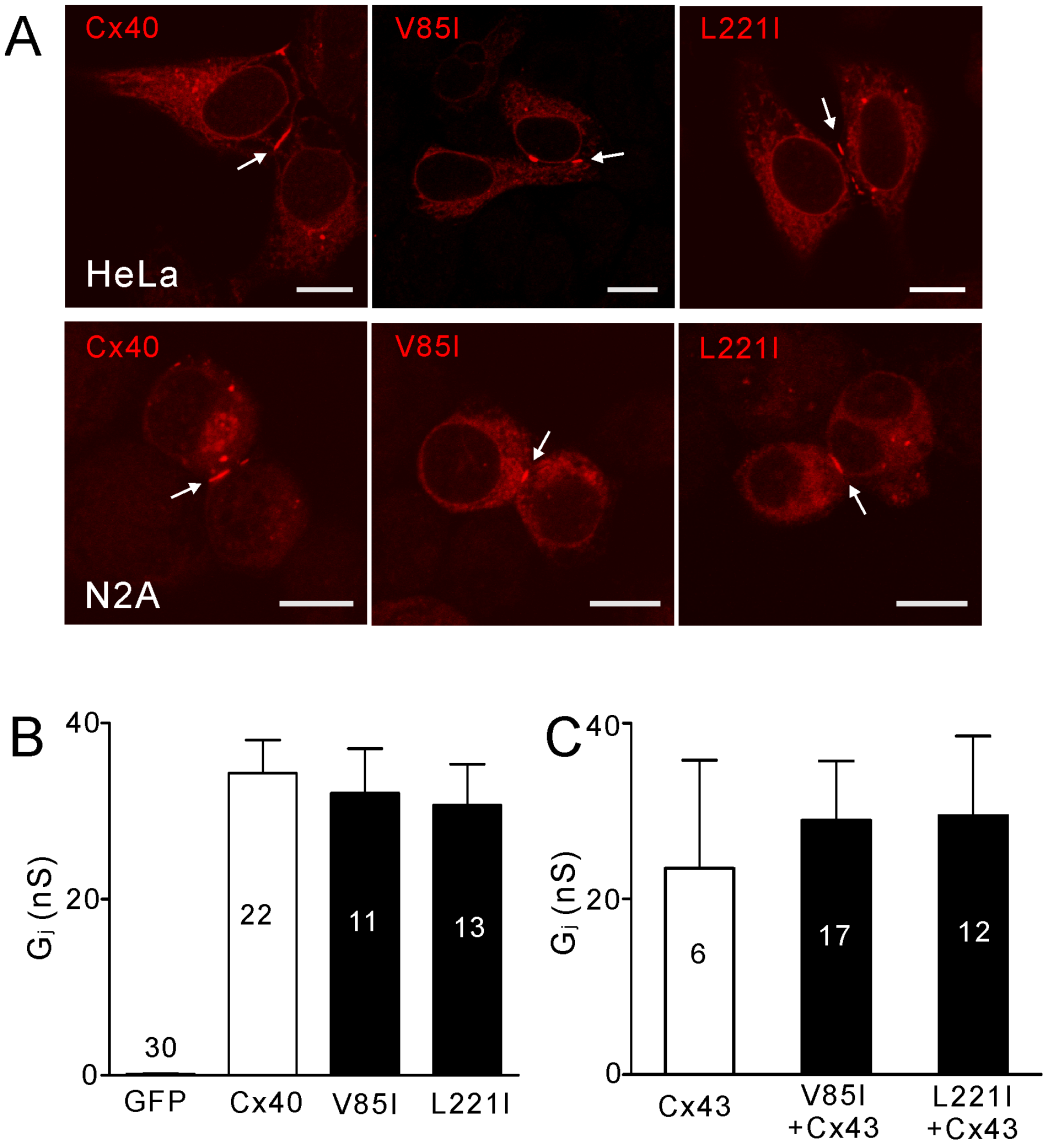
The localization and function of untagged homotypic Cx40 and Cx40-mutant gap junctions. (**A**) Representative confocal images showing the anti-Cx40 antibody localizations of untagged Cx40, V85I and L221I expressed in HeLa (top panels) and N2A (bottom panels) cells. In both cell lines, Cx40, V85I and L221I were all able to traffic to the cell-cell interface and form gap junction plaque-like structures. Scale bar = 10 µm. (**B**) There was no significant difference between the G_j_ of cell pairs expressing Cx40, V85I or L221I. (**C**) Co-expression of Cx40V85I-IRES-GFP or L221I-IRES-GFP with Cx43-mRFP (V85I+Cx43 or L221I+Cx43) in N2A cell pairs showed a similar G_j_ with cell pairs expressing Cx43-mRFP. The number of cell pairs are indicated on the bars.

Fusion of fluorescent proteins at the carboxyl terminus of connexins is very useful in determining the distribution and function of connexins in live cells. However, to verify our results obtained by using YFP-tagged Cx40, we also studied untagged Cx40 mutants using mutant-IRES-GFP constructs. We expressed V85I-IRES-GFP and L221I-IRES-GFP in HeLa and N2A cells. Anti-Cx40 antibody was used to reveal the localization of expressed Cx40 mutants. As shown in Fig. 4A, V85I and L221I showed a similar intracellular distribution pattern and both of them were able to reach cell-cell interfaces to form gap junction plaque-like structures, similar to that observed for wild-type Cx40, in both HeLa and N2A cells.

The coupling conductance (G_j_) of N2A cell pairs expressing either one of these mutants showed a similar level as that of Cx40-expressing cells (Fig. 4B). In contrast, GFP-expressing cells failed to display any gap junction coupling (Fig. 4B). Co-expression of either one of these mutants with Cx43 in N2A cell pairs did not show any change in the coupling conductance compared to that of cell pairs expressing Cx43 (Fig. 4C).

**Figure 5:**
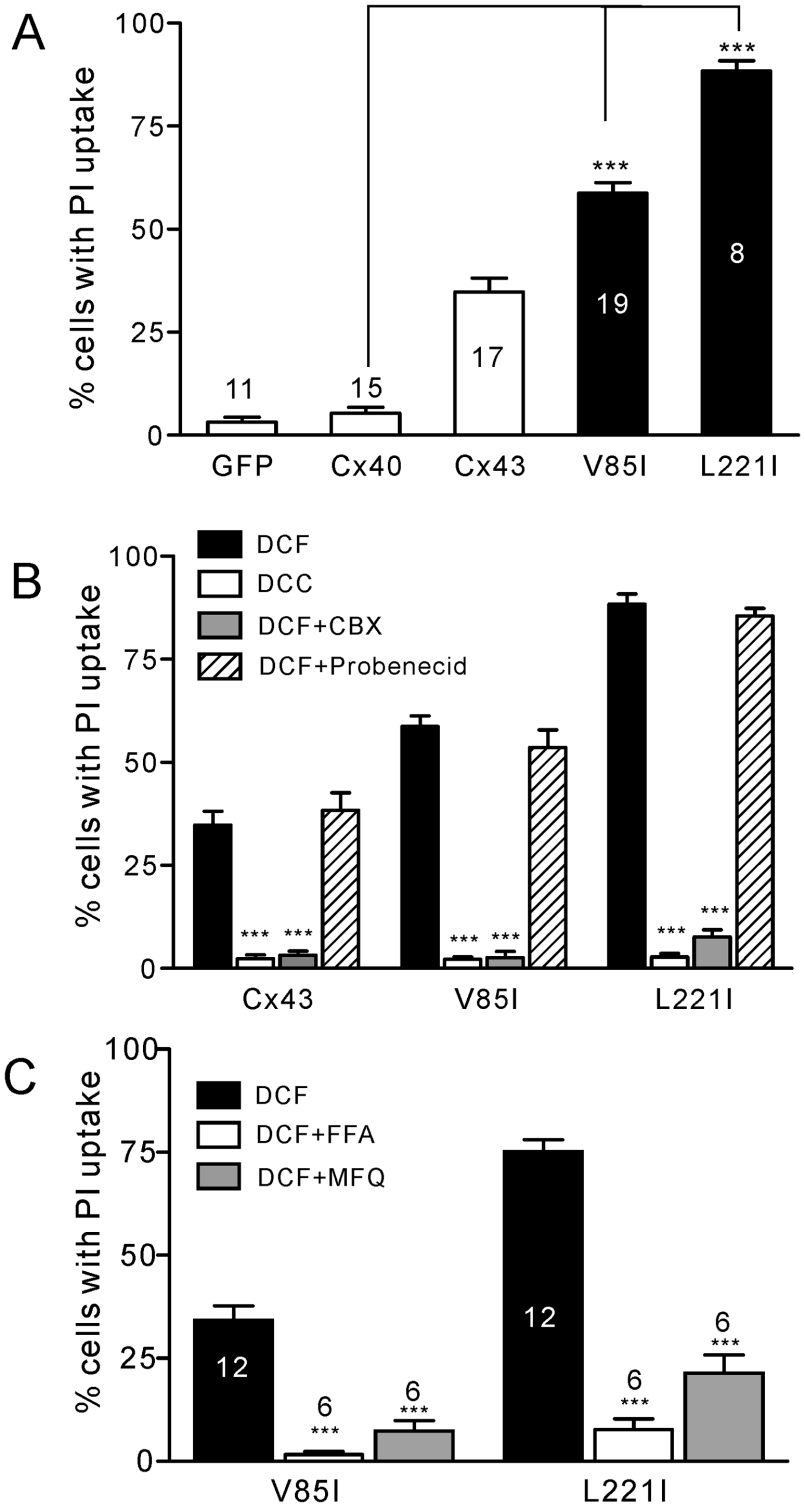
PI-uptake of untagged Cx40 and Cx40 mutants. (**A**) Untagged V85I and L221I showed a significant increase in PI-uptake compared to both wild-type Cx40 and Cx43 under the divalent cation-free (DCF) conditions. (**B**) The addition of divalent cations (DCC, open bars) or CBX (100 µM, gray bars) blocked the PI-uptake from cells expressing Cx43, V85I and L221I. However, the addition of the pannexin 1 channel blocker probenecid (200 µM, hatched bars) did not affect PI-uptake. (**C**) PI-uptake in cells expressing untagged V85I or L221I under DCF were significantly (P<0.001 in both cases) reduced by the addition of flufenamic acid (FFA, 50 µM) or mefloquine (MFQ, 25 µM). The total number of experiments are indicated on the bar.

PI-uptake was assessed the same way as described earlier by incubating HeLa cells in DCF-ECS. Both V85I and L221I showed a significantly higher level of PI-uptake than that of Cx40 (Fig. 5A). We also found that cells expressing these two mutants displayed higher levels of PI-uptake than that of wild-type Cx43 (Fig. 5A). Adding divalent cations (DCC) or CBX (100 µM) virtually eliminated PI-uptake, while the pannexin 1 channel blocker, probenecid (200 µM), failed to decrease PI-uptake (Fig. 5B). In addition, flufenamic acid (FFA, 50 µM) or mefloquine (MFQ, 25 µM) blocked the majority of PI-uptake in either V85I or L221I-expressing cells (Fig. 5C). These results confirmed that PI-uptake was due to undocked hemichannels and unlikely to be pannexin 1 channels.

**Figure 6:**
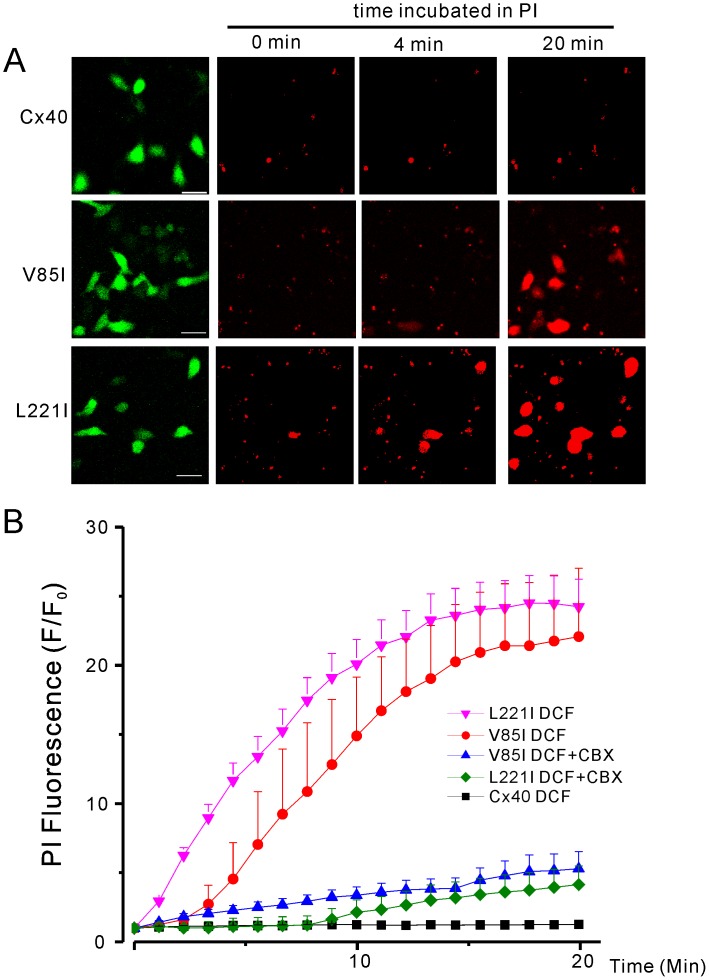
Time course of PI-uptake under the DCF conditions. (**A**) Representative confocal images of PI-uptake for HeLa cells transfected with wild-type Cx40 or Cx40 mutants (V85I or L221I). Time points of 0, 4 and 20 minutes of incubation with PI are displayed. During 20 minutes of incubation with PI, only cells expressing the V85I and L221I mutants showed PI uptake. Scale bar = 50 µm. (**B**) Ratio of current PI fluorescence intensity over the initial baseline fluorescence over a 20 minute incubation. L221I showed the fastest rate of PI-uptake, with V85I having a slightly slower rate of uptake, however, both L221I and V85I had similar levels of PI-uptake near the end of 20 minute incubation. The addition of 100 µM CBX blocked PI-uptake. Cx40 expressing cells failed to show any PI-uptake.

To evaluate the time course of PI-uptake during the incubation with DCF medium, we monitored the cellular PI-fluorescent level changes. L221I- and V85I-expressing cells showed a time-dependent increase in PI-uptake and saturated near the end of the 20 minute incubation (Fig. 6). V85I-expressing cells showed a slightly slower rate of PI-uptake within the first 10 minutes than that of L221I-expressing cells. However, they both reach a similar level of PI-uptake near the end of 20 minute-incubation. Addition of CBX (100 µM) virtually abolished the PI-uptake of mutant-expressing cells.

### Patch Clamp Recording of Putative Hemichannel Current in Mutant-expressing HeLa Cells

**Figure 7:**
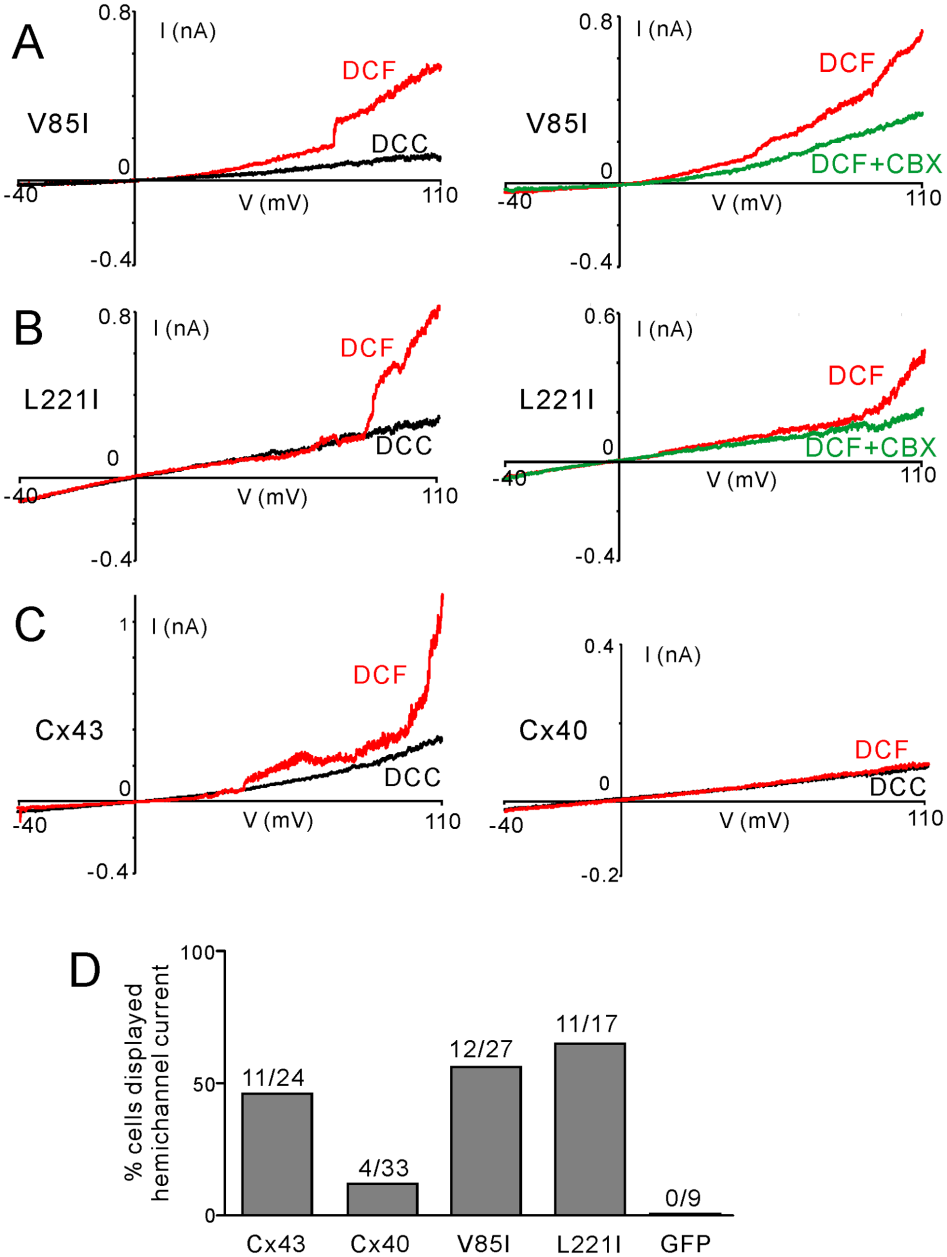
Putative hemichannel currents were increased in the HeLa cells-expressing AF-linked Cx40 mutants. Voltage clamp ramp protocol (−40 to 110 mV) was used to study currents under divalent cation containing saline (DCC, black traces) and the divalent-cation free saline (DCF, red traces). Putative hemichannel currents (the current amplitude differences between red and black traces) were observed in cells expressing AF-linked Cx40 mutants, V85I (A), L221I (B) and wild-type Cx43 (C, left panel). Most of Cx40-expressing cells failed to show the current (C, right panel). The DCF-dependent currents in the mutant-expressing cells were largely blocked by carbenoxolone (CBX, 100 µM, green traces). Bar graph summarized the percentages of cells displayed hemichannel current during the voltage ramp under DCF conditions (D). The connexin constructs expressed and the numbers of cells recorded are indicated. Note only 4/33 cells expressing Cx40 displayed putative hemichannel current (data not shown).

Voltage-clamp recording was used to study hemichannel current on HeLa cells expressing the Cx40 mutants and Cx40. A voltage ramp protocol induced an extra outward current in positive voltages (+20 mV or higher) after exchanging the saline (DCC) with DCF saline in the majority of mutant-expressing cells (Fig. 7A, B). The DCF-dependent currents in these V85I- or L221I-expressing cells were largely blocked by CBX (100 µM). Although the DCF-dependent currents were also observed in Cx43 or Cx40-expressing cells, the incidence was lower, especially in Cx40-expressing cells, where most of cells displayed no change in the voltage ramp-induced current in DCF (Fig. 7C, D). None of the GFP-expressing cells showed DCF-dependent current (Fig. 7D). These results support a model where the DCF-dependent and CBX-sensitive current are mediated, at least in part, by the mutant (or wild-type connexin) hemichannels. Both V85I and L221I increased the incidences of observing the hemichannel current compared to that of Cx40.

## Discussion

**Figure 8:**
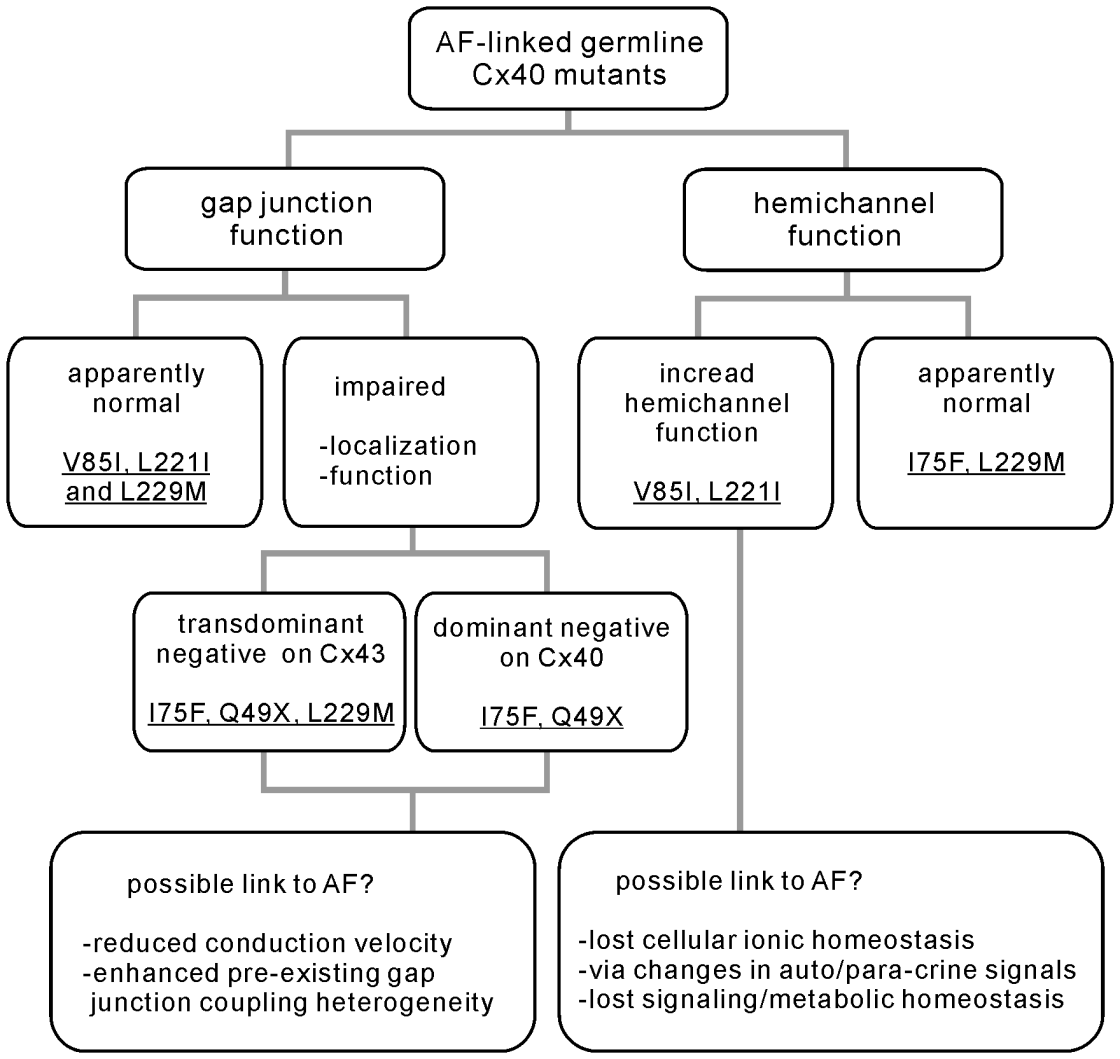
Overall summary of AF-linked germline Cx40 mutants. AF-linked germline Cx40 mutants have been shown to impair gap junction function via impaired localization (Q49X) or channel function (I75F). Dominant negative on Cx40 (Q49X and I75F) and/or transdominant negative actions on Cx43 were also observed (Q49X, I75F and L229M) [12], [15]. Present study showed that AF-linked Cx40 mutants, V85I and L221I, increased hemichannel function.

Here we studied two AF-linked germline mutations in the Cx40 gene. Our data indicated that V85I and L221I showed a statistically significant reduction in gap junction plaque formation at cell-cell interfaces. However, the functional inspection of the coupling conductance in N2A cell pairs expressing any of the mutants did not show a change from that of the wild-type Cx40, indicating that these mutants are unlikely to impair gap junction function. To further evaluate if there were any changes in the hemichannel function, we performed PI-uptake assays in the divalent cation-free medium. Cells expressing wild-type Cx40 did not show any PI-uptake, but V85I- and L221I-expressing cells showed pronounced PI-uptake. PI-uptake was eliminated or significantly reduced by the elevation of [Ca^2+^]_o_ or hemichannel blocking agents, carbenoxolone, FFA and MFQ, but was not blocked by probenecid, indicating that the PI-uptake is likely due to the opening of connexin hemichannels. Patch clamp recording also showed an increased incidence of the putative hemichannel current in the mutant-expressing cells during large membrane depolarizations. Lack of expression of pannexin 2 and 3 and no change in pannexin 1 expression with the expression of either Cx40 or L221I, indicating that pannexins are unlikely to play a major role in the observed hemichannel functions. The gain-of-function on hemichannels for these two AF-linked Cx40 mutants provided a completely novel mechanism for possible AF-pathogenesis. Our tests on several previously studied AF-linked germline Cx40 mutants suggested that a number of different mechanisms could link these mutants to AF, including impaired steady-state localization to the gap junction site, reduced/eliminated gap junction channel function or increased hemichannel function (Fig. 8). A detailed understanding of the AF-linked Cx40 mutants will be crucial in developing proper, effective strategies to treat AF.

Atrial fibrillation is the most common sustained cardiac arrhythmia and the prevalence is predicted to increase due to the aging of our population [20]. AF is characterized by rapid and irregular atrial activations, which are followed by uncoordinated and ineffective atrial contractions. This can lead to stagnant blood pooling in the atria and lead to thrombosis formation and is therefore a leading cause of embolic stroke [21]. Approximately 30% of AF patients have a form of AF that is not secondary to other cardiovascular problems, such as hypertension, heart failure or myocardial infarction, and this is known as lone or idiopathic AF [20], [22]. Although most of the Cx40 mutants show some sort of impairment in terms of forming functional gap junction channels, V85I and L221I show normal channel function when expressed alone or together with Cx43, but are gain of hemichannel function mutants. With this result comes the question of how this gain of hemichannel function can contribute to AF. Several possibilities associated with the opening of hemichannels may directly or indirectly change electrical properties of the cardiomyocytes, which could promote atrial arrhythmias. 1) Open hemichannels would allow for the inward and outward fluxes of Na^+^ and K^+^ ions according to their electrochemical gradient, respectively, the result of which is membrane depolarization. Transient membrane depolarization brings the cell closer to/over the threshold of firing an action potential, while sustained depolarization may lead to substantial Na^+^ channel inactivation, which can reduce the excitability of cardiomyocytes and lead to a slower conduction velocity. Both actions could increase the heterogeneity of cardiomyocyte excitability, increasing the susceptibility to arrhythmias [23]. 2) Open hemichannels may lead to the release of ATP to the extracellular space. The released ATP could act in an autocrine and paracrine manner to cause intracellular Ca^2+^ wave propagation via purinergic receptors [3]. 3) It is well-documented that most of the characterized gap junction hemichannels are large enough to allow leakage of small signaling and nutrient/metabolic molecules. The mutant Cx40 hemichannels may provide a passage to lose some of these important molecules, which may be critical for normal cardiomyocyte function. All of the above possibilities have the potential to lead to abnormal activities of the cardiomyocytes and may play a role in generating arrhythmias in patients carrying these mutations. It is noted that in normal physiological conditions these mutant hemichannels are unlikely to be opened, however, our data indicate that both reduction of [Ca^2+^]_o_ and large membrane depolarization (e.g. during the peak of action potentials) can promote the mutant hemichannel opening.

Previous studies on AF-linked gap junction mutants are focused on the localization and function of gap junction channels. Cx40 mutants, P88S and Q49X, as well as the Cx43 mutant, G60S, showed impaired trafficking to the cell surface [11], [15], [24], while Cx40 mutants, I75F, A96S, L229M and G38D did not show any alterations in their localization, but displayed various degrees of reduction of the GJ coupling conductance [11], [12]. In any case, the GJ function is impaired via different underlying mechanisms. It is predicted that the connexin mutants with impaired localization would also likely eliminate/reduce their hemichannel function. However, it is not known whether hemichannel function is reduced, increased or unchanged in those mutants with impaired gap junction function because other disease-linked Cx43 and Cx30 mutants with GJ impairment can increase [25] or decrease hemichannel function [26]. Here we tested AF-linked Cx40 mutants, Q49X, I75F and L229M, PI-uptake in DCF conditions and found no measurable increase in PI-uptake in our experimental conditions. At this time we are unable to identify if there is a decrease in the hemichannel function for these mutants due to the fact that we did not observe any substantial PI-uptake in wild-type Cx40 hemichannels. This is in contrast to the results seen with V85I or L221I, which show no obvious defect in gap junctional conductance when expressed alone or co-expressed with wild-type Cx43; however they showed prominent PI-uptake under DCF conditions, indicating a gain-of-hemichannel function.

Although many Cxs have been investigated for their hemichannel function, a number of them have not yet been properly characterized to our knowledge, including Cx40. Our current study is the first functional study of wild-type Cx40 and its mutant hemichannels. One study by Allen et al. (2011), used atomic force microscopy to evaluate the three-dimensional molecular topology and calcium-dependent conformational changes of Cx40 hemichannels [27]. They demonstrated that at low [Ca^2+^]_o_ (<10 µM), Cx40 hemichannels showed surface openings. The increase of external Ca^2+^ concentration closed most of the Cx40 hemichannels, suggesting that Ca^2+^ ions cause conformational rotation of Cx40 subunits, which act to close the pore. They also noted that the addition of EDTA, which acts as a Ca^2+^ chelator in a similar way to EGTA, led to Cx40 hemichannel opening [27]. Although this study reported the structural hemichannel opening of Cx40, they did not study if the morphological changes observed can translate into functional changes of Cx40 hemichannels. Here we provide experimental evidence that eliminating extracellular Ca^2+^, Mg^2+^ and a large membrane depolarization may lead to the opening of Cx40 hemichannels in a small fraction of cells (4/33, Fig. 7D), however, this open state may be too small to allow the large fluorescent dye, PI, to pass through.

There is an apparent contradiction in our electrophysiological data (both DCF and substantial membrane depolarization are required to activate the hemichannel current) and the PI-uptake data (DCF at resting membrane potential is sufficient to show PI-uptake via hemichannels). However, it is important to note that there is a key difference in temporal domain between these two hemichannel-mediated processes: hemichannel current is rapid and observable in milliseconds time scale, while the PI-uptake is much slower to develop, requires 15 minutes to reach a saturation level. It is possible that each hemichannel may have two gates (could be those described slow ‘loop’-gate on the extracellular end and the fast V_j_-gate on the cytosol end of a hemichannel) [28], hemichannel currents could only be recorded when both gates are open, e.g. during depolarized membrane potentials in DCF. While these two gates could be opened sequentially to transfer PI dye at resting membrane potential, but they might rarely open both gates simultaneously to produce hemichannel current. An alternative possibility could be that the hemichannel could be opened at resting membrane potential, but the probability of opening is too low to be recorded during the short ramp protocols. Future systematic studies may help to understand the molecular mechanisms of hemichannel gating of these AF-linked Cx40 mutants.

Specific increases in connexin hemichannel function have been associated with human disease-linked mutations in several connexin genes. For example, two Clouston syndrome-linked Cx30 mutants, G11R and A88V, have been previously reported to increase hemichannel function while maintaining normal gap junction function. Under physiological conditions, both G11R and A88V show increased ATP release via hemichannels compared to wild-type Cx30 [29]. The Cx32 mutation S85C, which is associated with X-linked Charcot-Marie-Tooth (CMTX) disease, has shown large hemichannel-mediated voltage-dependent currents that are not seen with wild-type Cx32 [30]. Another CMTX Cx32 mutation, F235C, also forms leaky hemichannels that contribute to a very severe neuropathy [31]. Leaky hemichannels have also been reported in Cx26 mutations linked to keratitis-ichthyosis-deafness syndrome, G45E [32] and A40V [33]. Both mutants exhibit normal gap junction function, but A40V has altered extracellular Ca^2+^ regulation leading to a higher hemichannel open probability and G45E shows increased permeability to Ca^2+^ [34]. Finally, three oculodentodigital dysplasia-linked Cx43 mutants, I31M, G138R and G143S, all showed greater than a 2-fold increase in hemichannel activity compared to wild-type Cx43 as demonstrated by an increase in ATP release [35]. It should be noted that the G138R mutant also impaired GJ function in addition to increasing hemichannel activity. Our current study provides the first demonstration of Cx40 mutant hemichannel function, giving us new insights into the etiology of lone AF.

## Conclusion

Two germline Cx40 mutations, V85I and L221I, were identified and each was linked to a Chinese family suffering from lone AF. These mutations showed impaired trafficking to the cell surface, but did not significantly impair gap junction function. Wild-type Cx40 did not show any PI-uptake, signifying a likely closed hemichannel under our experimental conditions. Both the Cx40 mutants showed significantly increased PI-uptake and hemichannel current compared to wild-type Cx40, suggesting a gain of hemichannel function. These findings demonstrate the first functional study of Cx40 hemichannels and also describe a novel mechanism by which Cx40 mutants may contribute to the pathogenesis of lone AF.
